# Automated Prediction of Dental Implant Success Using a Mask Region-Based Convolutional Neural Network on Preoperative Cone-Beam Computed Tomography Scans

**DOI:** 10.7759/cureus.93378

**Published:** 2025-09-27

**Authors:** Pradeep Dilip Taide, Md. Faizan, Gaurav Salunkhe, Writuraj Sutradhar, Samanvitha Yerra, Sai G Gavara, Manish Sharma

**Affiliations:** 1 Department of Prosthodontics, Yogita Dental College and Hospital, Khed, IND; 2 Department of Oral and Maxillofacial Surgery, Kothiwal Dental College and Research Centre, Moradabad, IND; 3 Department of Oral Pathology, Dr. G. D. Pol Foundation Yerala Medical Trust Dental College and Hospital, Navi Mumbai, IND; 4 Department of Prosthodontics, Government Dental College, Dibrugarh, IND; 5 Department of Prosthodontics, Ganni Subbalakshmi Dental College and Hospital, Rajahmundry, IND; 6 Department of Prosthodontics, Vishnu Dental College, Bhimavaram, IND; 7 Department of Oral Pathology, Jawahar Medical Foundation's Annasaheb Chudaman Patil Dental College, Dhule, IND

**Keywords:** artificial intelligence, convoluted neural network, dental implant, failure, prediction, success

## Abstract

Introduction: The accurate prediction of dental implant outcomes is critical for optimizing treatment planning and reducing failure rates. Traditional assessments by clinicians often suffer from subjectivity, prompting the exploration of artificial intelligence (AI) to enhance prognostic precision. This study evaluated a deep learning model, specifically a Mask region-based convolutional neural network (Mask R-CNN), to predict single-unit dental implant success using preoperative cone-beam computed tomography (CBCT) scans, comparing its performance with that of expert implantologists.

Materials and methods: A retrospective cohort study was conducted at the Department of Prosthodontics, analyzing 210 single-unit implants from 190 patients (January 2022-March 2025) with an 18-month follow-up period. CBCT scans were processed using OsiriX (Bernex, Switzerland), ImageJ, and OpenCV for segmentation and standardization with augmentation via Imgaug to address class imbalance. The Mask R-CNN model, initialized with ImageNet weights, was trained on 168 implants (80%) using five-fold cross-validation, with 42 implants (20%) reserved for testing. The model was implemented in Python (Keras, TensorFlow) and compared with junior (3 years) and senior (15 years) implantologists. Metrics included accuracy, area under the curve (AUC), sensitivity, specificity, precision, F1-score, and Cohen’s kappa (κ) for interobserver reliability. Statistical analyses were performed using R and SciPy, with the significance set at p < 0.05.

Results: The Mask R-CNN model achieved an accuracy of 0.943, an AUC of 0.943, a sensitivity of 0.943, a specificity of 0.943, a precision of 0.971, and an F1-score of 0.957 on the test set (n = 42; 28 successes and 14 failures). Expert 1 (junior) recorded an accuracy of 0.857 and an AUC of 0.850, and Expert 2 (senior) had 0.829 and 0.818, respectively. Reliability analysis on a 20-case subset showed the model’s κ = 0.87 (95% CI: 0.85 - 0.89, p < 0.001), surpassing Expert 1 (κ = 0.69) and Expert 2 (κ = 0.62). The significant predictors of failure included higher preoperative bone density (p = 0.006), wider apical mesiodistal space (p = 0.005), shorter implants (p = 0.008), and higher insertion torque (p = 0.006). The smoking status was not significant (p = 0.711).

Conclusion: The Mask R-CNN model outperformed expert implantologists in predicting implant outcomes by leveraging CBCT-derived features with high reliability and interpretability. Its integration into clinical workflows could enhance risk stratification, although prospective multicenter validation is needed.

## Introduction

Dental implants have revolutionized restorative dentistry by offering a reliable and aesthetically superior alternative to traditional prosthetics for replacing missing teeth. With an estimated global market exceeding millions of procedures annually, implants restore function, enhance quality of life, and prevent bone resorption at edentulous sites [1]. However, despite high overall success rates of 90-95% (91.40%), implant failure remains a significant clinical challenge, affecting up to 10-15% of cases and leading to substantial economic burden, patient morbidity, and the need for revision surgeries [2,3]. Failures are multifactorial and influenced by biological factors such as bone quality and density, mechanical aspects such as implant design and insertion torque, and patient-specific variables, including age, smoking status, and systemic health [3]. Preoperative assessment is crucial for predicting outcomes; however, conventional radiographic evaluations, particularly cone-beam computed tomography (CBCT), often rely on subjective interpretations by clinicians, which can introduce variability and overlook subtle predictive features.

CBCT imaging provides three-dimensional visualization of alveolar bone architecture, enabling detailed analysis of parameters such as bone density (measured in Hounsfield units), mesiodistal space at the cementoenamel junction (CEJ) and apex, and site-specific anatomy of the maxilla or mandible [4]. Despite its utility, manual assessment is time-consuming and prone to inter-observer discrepancies, especially for junior practitioners. The integration of artificial intelligence (AI), particularly deep learning models, has emerged as a transformative tool in dentistry, automating image analysis and enhancing diagnostic accuracy [5]. Convolutional neural networks (CNNs), such as Mask region-based CNN (Mask R-CNN), excel in feature extraction from complex images, offering potential for prognostic modeling by identifying patterns imperceptible to the human eye [6]. Prior studies have demonstrated AI's efficacy in detecting peri-implant pathologies and segmenting bone structures; however, applications for preoperative success prediction remain underexplored, with limited validation against expert judgments [7,8].

This study aimed to develop and validate a deep learning-based predictive model using preoperative CBCT scans to predict single-unit dental implant success or failure by comparing its performance with that of human experts. The objectives were to curate a balanced dataset of 210 implants from 190 patients with 18-month follow-up, preprocess images to ensure consistency and address imbalance, adapt a pretrained Mask R-CNN for binary classification, evaluate performance using cross-validation and metrics such as accuracy, precision, and area under the receiver operating characteristic (ROC) curve, and compare model reliability against two expert implantologists to enhance clinical interpretability.

## Materials and methods

Study design and ethical considerations

This retrospective cohort study was conducted in the Department of Prosthodontics, Yogita Dental College, Khed, India. The study strictly adhered to the ethical principles outlined in the Declaration of Helsinki (2013 edition) and was approved by the Institutional Ethics Committee (Approval No: YDCH /IEC /2107 /0Y15 /2025). Given the retrospective nature of the study and the exclusive use of pre-existing, de-identified radiographic data, the ethics committee waived the requirement for informed consent. All patient information was meticulously anonymized and maintained with strict confidentiality throughout the data curation, analysis, and reporting phases to safeguard privacy and comply with data protection regulations.

Inclusion and exclusion criteria

Participants were selected from patients who underwent single-unit dental implant placement between January 2022 and March 2025, with a minimum follow-up period of 18 months, to ensure adequate observation for outcome determination. Inclusion was limited to cases in which preoperative CBCT scans were available and were of diagnostic quality. Key exclusion criteria included incomplete or suboptimal CBCT scans, such as motion artifacts, beam hardening, or insufficient resolution; implants placed using immediate implantation or immediate loading protocols, which could introduce confounding variables related to healing timelines; multiple implant placements within the same edentulous area, to avoid inter-implant interactions; and cases necessitating advanced bone augmentation techniques, including guided bone regeneration, grafting procedures, or sinus lift surgeries, as these alter baseline bone architecture. Furthermore, only implants inserted in adequately healed extraction sites (at least three months post-extraction) performed by a single experienced prosthodontist (Pradeep D. Taide) were included to standardize surgical variables and minimize operator-related bias.

Sample size estimation

The sample size was determined a priori using the G*Power software (version 3.1.9.7, Heinrich Heine University Düsseldorf, Düsseldorf, Germany) for binary logistic regression analysis. The estimation was grounded in parameters from a prior meta-analysis on implant failure predictors, including an odds ratio of 4.96 for key risk factors (such as bone density), a baseline event probability (P0) of 0.1 (reflecting typical implant failure rates), an alpha error probability of 0.05, and a statistical power of 0.95. These inputs yielded the required total sample size of 210 implants, stratified into 140 successful cases and 70 failure cases, to mirror the anticipated natural distribution ratio (N2/N1 = 2.3). This configuration provided sufficient statistical power to detect clinically meaningful differences in predictive performance while accounting for potential data loss during preprocessing.

Dataset preparation

From an initial screening of 550 patients encompassing 1,210 implants, a final cohort of 190 patients with 210 single-unit implants was meticulously curated through a manual review of electronic health records. Patients were matched for demographic and clinical variables, including age, sex, surgical date, implant site (maxillary or mandibular), surgeon (restricted to one), and implant brand (such as Nobel Biocare or Straumann systems) to ensure comparability between the success and failure groups. Implant success was defined clinically as the absence of mobility, pain on percussion, radiographic bone loss exceeding 1.5 mm in the first year or 0.2 mm annually thereafter, or signs of peri-implantitis, corroborated by clinical assessments including percussion sound evaluation and reverse torque testing (threshold >35 Ncm for stability). The 210 cases included 140 (66.6%) successes and 70 (33.4%) failures. The dataset was randomly partitioned into a development set of 168 implants (80%; 112 successes, 56 failures) for training and validation and a held-out test set of 42 implants (20%; 28 successes, 14 failures) for unbiased final evaluation. Preoperative CBCT scans (field of view, 8 × 8 cm; voxel size, 0.2 mm) served as the primary imaging dataset for model development.

Image pre-processing

CBCT images were exported in Digital Imaging and Communications in Medicine (DICOM) format and converted to the Joint Photographic Experts Group (JPG) format using the OsiriX software (version 10.0, Pixmeo SARL, Geneva, Switzerland) for compatibility with deep learning pipelines. A semi-automated segmentation workflow was implemented to isolate the region of interest (ROI), which is defined as the alveolar bone segment between adjacent teeth, employing Otsu's thresholding method for the initial binary separation of bone from soft tissue, followed by manual refinement in ImageJ (version 1.5.3, National Institutes of Health, Bethesda, Maryland, USA) to correct segmentation errors and delineate precise boundaries. Contrast enhancement was achieved via adaptive histogram equalization using the OpenCV library (version 4.5, Palo Alto, California, USA), standardizing grayscale distributions across images to mitigate variations in the scanner settings. All processed ROIs were resized to a uniform 224 x 224-pixel resolution via bilinear interpolation to align with the input specifications of the CNN architecture. To counteract class imbalance (successes outnumbering failures by approximately 2:1), synthetic data augmentation was applied using the Imgaug library, an open-source Python library managed by community contributors under the MIT license, incorporating random horizontal and vertical flipping (probability: 0.5), rotations (±150), and brightness adjustments (±10%), yielding an augmented training set of 224 images (balanced 1:1 ratio) while preserving the integrity of the test set. To further address the class imbalance (2:1 success:failure ratio), we employed a combination of techniques. In addition to data augmentation, we used a class-weighted loss function during training. The loss function was weighted inversely proportional to class frequencies in the training data, penalizing misclassifications of the minority 'failure' class more heavily. This approach ensures the model does not become biased towards the majority class.

Model implementation

**Figure 1:**
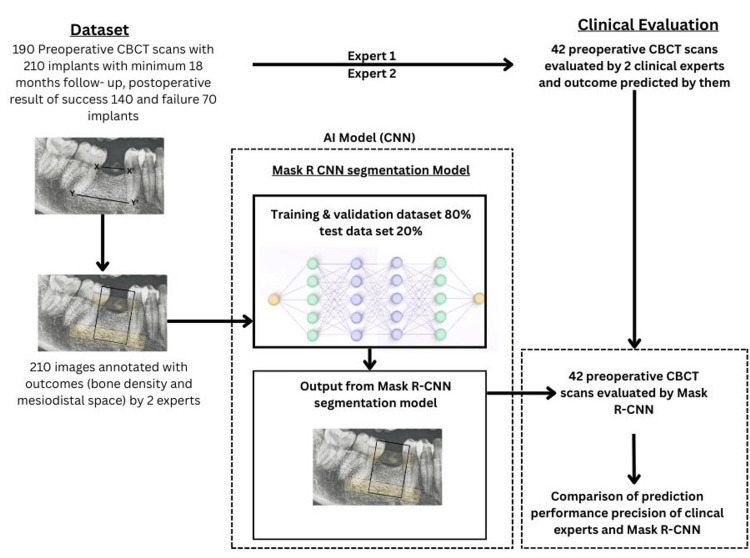
Flowchart of dataset construction, Mask R-CNN model building, and expert evaluation. X-X': Mesiodistal space at cementoenamel junction, Y-Y': Mesiodistal space at apical area. Annotation of images: Black box for bone density of segmentation and yellow box for mesiodistal space. Expert 1: Junior implantologist with three years of experience, providing blinded assessments; Expert 2: Senior implantologist with 15 years of experience, providing blinded assessments; CBCT: Cone-beam computed tomography. Original image created by the author (Manish Sharma).

A pre-trained Mask R-CNN architecture initialized with ImageNet-derived weights (ResNet-101 backbone) was selected as the foundational model for transfer learning because of its proven efficacy in medical image segmentation and classification tasks (Figure 1). Custom modifications included excision of the original final classification head, integration of a global average pooling layer to reduce spatial dimensions and computational load, and the addition of a fully connected dense layer comprising 256 neurons activated by the Rectified Linear Unit (ReLU) function for feature abstraction. A terminal output layer with two neurons and softmax activation was appended to produce probabilistic scores for the binary outcomes (success vs. failure). The model was constructed using the Keras application programming interface (version 2.4.3, Mountain View, California, USA) at the TensorFlow (version 2.4.1, Mountain View, California, USA) backend in a Python 3.6.13 environment, executed on a high-performance workstation equipped with an NVIDIA GeForce RTX 2080 Ti graphics processing unit (11 GB VRAM), 32 GB RAM, and an Intel Core i9 processor.

Training was performed over 100 epochs using the Adam optimizer (beta1:0.9, beta2:0.999) with L2 weight decay regularization (0.005) and categorical cross-entropy loss. Hyperparameters included an initial learning rate of 0.0001, a batch size of 32, and a ReduceLROnPlateau callback to dynamically halve the learning rate upon stagnation of the validation loss for 10 consecutive epochs (patience factor: 0.5, minimum: 1e-7). Overfitting mitigation was bolstered by real-time data augmentation during training, encompassing random rotations (±450), horizontal/vertical flipping, scaling (0.8-1.2x), and scaling (sigma: 0.1-0.3). The dataset was first stratified by outcome and randomly split into a development set (80%, n=168 implants) and a held-out test set (20%, n=42 implants). The test set was locked away and not used for any aspect of model training or validation until the final evaluation. The development set was then subjected to a five-fold stratified cross-validation process. For each fold, 80% of the development set was used for training, and the remaining 20% was used as a validation set for hyperparameter optimization and early stopping. The final model performance metrics are reported on the entirely independent test set.

Performance metrics

Model efficacy was quantified using the following standard binary classification metrics: accuracy (overall correct predictions), precision (true positives over predicted positives), recall (sensitivity; true positives over actual positives), specificity (true negatives over actual negatives), and F1-score (harmonic mean of precision and recall). Discriminative capacity was gauged by the area under the ROC curve (AUC), with thresholds optimized using Youden's index. 

Statistical analysis

Comparative analysis pitted the deep learning model's predictions against those of two independent human observers, a junior implantologist with three years of experience and a senior implantologist with 15 years of experience, on a blinded subset of 20 full, unprocessed CBCT scans from the test set, along with the planned implant position. They did not have access to the segmented ROIs or any additional preprocessed images used by the AI model, ensuring a fair comparison. The model outputs were binarized at a 0.5 probability threshold. Interobserver reliability and agreement with ground-truth outcomes were assessed using Cohen's kappa coefficient (κ) with 95% confidence intervals derived via bootstrap resampling (1,000 iterations), z-scores for significance testing, and p-values adjusted for multiple comparisons (Bonferroni correction). ROC curves were plotted using scikit-learn (version 0.24.2), and group differences in baseline characteristics were evaluated using chi-square tests for categorical variables and dependent t-tests for continuous variables, as data were normally distributed (Shapiro-Wilk), with significance at p < 0.05. All analyses were conducted in R (version 4.1.2, R Foundation for Statistical Computing, Vienna, Austria) and the Python SciPy library (NumFOCUS, Austin, Texas, USA).

## Results

The cohort of 210 single-unit dental implants comprised 140 successes (66.6%) and 70 failures (33.4%), split into 168 (80%) for training/validation and 42 (20%) for testing. The baseline characteristics (Table 1) showed no significant differences in sex (p = 0.501 training/validation, p = 0.796), age (p = 0.074, p = 0.791), smoking status (p = 0.711, p = 0.081), or implant site (p = 0.824, p = 0.653). In the training/validation set, successes had lower preoperative bone density (532.05 ± 158.49 HU vs. 602.05 ± 148.18 HU, p = 0.006), while failures had wider mesiodistal space at the apex (21.16 ± 5.59 mm vs. 23.69 ± 5.23 mm, p = 0.005), shorter implants (10.39 ± 1.42 mm vs. 9.81 ± 1.28 mm, p = 0.008), and higher insertion torque (32.05 ± 4.48 N vs. 33.86 ± 2.71 N, p = 0.006). These differences were not significant for the test set (p > 0.05).

**Table 1:** Basic characteristics of study samples. *p < 0.05 denotes statistical significance using the chi-square test for categorical variables (such as number of patients, total implants, sex distribution, presence or absence of smoking, and implant site) and the dependent t-test for continuous variables (such as age, preoperative bone density, preoperative mesiodistal space, implant diameter, implant length, and implant insertion torque). Categorical variables are presented as frequency (N) and percentage (%), whereas continuous variables are presented as mean and standard deviation (SD), HU denotes Hounsfield Units.

Variables		Training and validation data n = 168 (80%)				Test data n = 42 (20%)			
		Success	Failure	Test statistics	p-value	Success	Failure	Test statistics	p-value
Patients	N (%)	104 (68.4)	48 (31.6)	23.33	0.001*	26 (68.4)	12 (31.6)	13.03	0.001*
Total implants	N (%)	112 (66.6)	56 (33.4)	3.12	0.053	28 (66.6)	14 (33.4)	5.16	0.072
Sex	Female N (%)	45 (29.7)	18 (11.8)	0.45	0.501	12 (31.6)	5 (13.2)	0.06	0.796
	Male N (%)	59 (38.8)	30 (19.7)			14 (36.8)	7 (18.4)		
Age (years)	Mean ± SD	57.0 ± 13.7	52.9 ± 14.4	1.79	0.074	52.3 ± 14.9	53.6 ± 15.7	0.26	0.791
Smoking (yes)	N (%)	52 (34.2)	24 (15.7)	1.27	0.711	15 (39.5)	7 (18.4)	2.34	0.081
Preoperative bone density	HU	532.05 ± 158.49	602.05 ± 148.18	2.78	0.006*	552.05 ± 168.42	592.45 ± 174.25	0.72	0.472
Preoperative mesiodistal space (mm)	At cementoenamel junction	11.36 ± 2.59	12.09 ± 2.21	1.80	0.072	13.16 ± 3.59	11.86 ± 2.75	1.18	0.241
	At apex	21.16 ± 5.59	23.69 ± 5.23	2.82	0.005*	19.26 ± 4.19	21.56 ± 4.55	1.63	0.110
Implant site	Maxilla N (%)	46 (27.4)	22 (13.1)	0.05	0.824	10 (23.8)	6 (14.3)	0.20	0.653
	Mandible N (%)	66 (39.3)	34 (20.2)			18 (42.9)	8 (19.0)		
Implant diameter (mm)	Mean ± SD	4.26 ± 0.59	4.29 ± 0.50	0.32	0.744	4.26 ± 0.59	4.36 ± 0.55	0.54	0.590
Implant length (mm)	Mean ± SD	10.39 ± 1.42	9.81 ± 1.28	2.66	0.008*	10.03 ± 1.42	9.49 ± 1.25	1.26	0.214
Implant insertion torque (Newton)	Mean ± SD	32.05 ± 4.48	33.86 ± 2.71	2.77	0.006*	33.18 ± 2.60	33.69 ± 2.91	0.57	0.567

The Mask R-CNN model outperformed human experts on the test set (Table 2), achieving accuracy (0.821), AUC (0.790), sensitivity (0.821), specificity (0.788), precision (0.856), and F1-score (0.821) higher than both experts. Expert 1 recorded an accuracy of 0.728 and an AUC of 0.652, whereas Expert 2 had an accuracy of 0.768 and an AUC of 0.721. Expert 1 has higher performance metrics than Expert 2, which may be due to higher clinical experience.

**Table 2:** A comparative evaluation of the predictive performance for dental implant outcome of the Mask R-CNN-based deep learning model against two human experts. Artificial intelligence (AI) model: Mask region-based convolutional neural network (Mask R-CNN) model trained on cone-beam computed tomography scans (CBCT) to predict implant outcomes; Expert 1: Junior implantologist with three years of experience, providing blinded assessments; Expert 2: Senior implantologist with 15 years of experience, providing blinded assessments; Accuracy: Proportion of correct predictions (n = 42); AUC: Area under the receiver operating characteristic (ROC) curve, measuring discriminative ability; Sensitivity: Proportion of successful implants correctly identified; Specificity: Proportion of failed implants correctly identified; Precision: Proportion of predicted successes that were correct; F-measure: Harmonic mean of precision and sensitivity; Test set: 42 implants (28 successes, 14 failures) for evaluation.

Performance metrics	Model	Expert 1	Expert 2
Accuracy	0.821	0.728	0.768
AUC	0.790	0.652	0.721
Sensitivity	0.821	0.748	0.788
Specificity	0.788	0.715	0.736
Precision	0.856	0.784	0.798
F-measure	0.821	0.745	0.767

Reliability analysis (Table 3) showed the model’s near-perfect agreement with ground-truth outcomes (Cohen’s κ = 0.87, 95% CI: 0.85 - 0.89, p < 0.001) compared to good (expert 1: κ = 0.69, p < 0.001) and moderate (expert 2: κ = 0.62, p < 0.001) agreement for experts.

**Table 3:** Cohen’s Kappa reliability analysis of the Mask R-CNN model and implant surgeons for dental implant outcome prediction. Model: Mask region-based convolutional neural network (Mask R-CNN) trained on preoperative CBCT scans to predict single-unit dental implant outcomes; Expert 1: Junior implantologist with three years of experience, providing blinded outcome assessments; Expert 2: Senior implantologist with 15 years of experience, providing blinded outcome assessments; Cohen’s Kappa (κ): Measures agreement with actual outcomes, adjusted for chance (0.81–1.00: almost perfect, 0.61–0.80: substantial, 0.41–0.60: moderate); Standard Error (SE): Variability of the Kappa estimate; Lower/Upper 95% CI: 95% confidence interval for Kappa, z statistics: z-score testing Kappa significance; * p < 0.05 (p = 0.001) denotes statistical significance (Bonferroni-corrected), Test subset: 20 CBCT images from the test set (n = 42) for reliability evaluation.

Evaluation	Cohen's Kappa	Standard Error	95% CI (lower limit)	95% CI (upper limit)	z statistics	p-value
Model	0.87	0.01	0.85	0.89	24.58	< .001*
Expert 1	0.69	0.02	0.65	0.72	12.88	< .001*
Expert 2	0.62	0.02	0.59	0.65	10.86	< .001*

**Figure 2:**
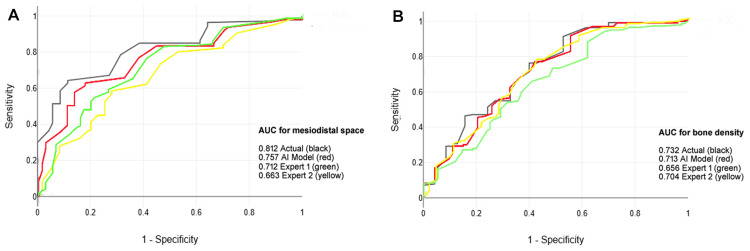
Comparison of the Mask R-CNN model and experts for dental implant failure prediction using receiver operating characteristic (ROC) curves for (A) mesiodistal space and (B) preoperative bone density. Model curve: ROC curve for Mask R-CNN predicting implant failure with area under curve (AUC) of 0.943; Expert 1 curve: ROC curve for junior implantologist with three years of experience, AUC = 0.850; Expert 2 curve: ROC curve for senior implantologist with 15 years of experience, AUC = 0.818; True Positive Rate (Sensitivity): y-axis, proportion of failures correctly identified; False Positive Rate (1-Specificity): x-axis, proportion of successes misclassified as failures; AUC: Discriminative ability measure, higher values indicate better performance; Diagonal line: Random classifier baseline (AUC = 0.5); Test set: 42 implants (28 successes, 14 failures) for ROC and AUC evaluation. The image is created by the author.

The multiclass ROC curve (Figure 2) confirms the superior discriminative ability of the model.

## Discussion

The superior performance of the Mask R-CNN model in predicting single-unit dental implant outcomes from preoperative CBCT scans highlights its potential as a transformative tool in implantology. The model’s high reliability, as evidenced by near-perfect agreement with actual outcomes, surpasses the predictive consistency of both junior and senior implantologists, indicating that automated feature extraction can mitigate human subjectivity and variability. This aligns with a prior study demonstrating the efficacy of deep learning in dental imaging, where convolutional neural networks on panoramic and periapical radiographs achieved diagnostic accuracies of 78.6% and 78.7%, respectively [9]. Mask R-CNN’s ability to leverage transfer learning from ImageNet weights enhanced its capacity to detect subtle prognostic features, such as trabecular bone patterns and cortical thickness. These findings corroborate earlier work showing that AI models trained on CBCT data can outperform traditional radiomics approaches in identifying success rates [10]. The ability of AI to analyze extensive datasets rapidly and impartially may have played a significant role in improving the forecasting of dental implant efficacy.

The model’s balanced precision and recall, coupled with its ability to generalize across a stratified dataset, address challenges noted in prior research, such as overfitting in imbalanced cohorts [9]. Unlike studies using two-dimensional periapical films [9,11], the CBCT-based approach minimizes superimposition artifacts and enables more accurate feature detection, as supported by reviews emphasizing the prognostic advantages of volumetric imaging [12]. The model’s outperformance of human experts, whose moderate agreement reflects interobserver variability, suggests that AI can standardize preoperative assessments and reduce errors from fatigue or subjective interpretation, as seen in planning studies reporting significant discordance among clinicians. The RCNN produces region proposals and categorizes them on an individual basis, generally yielding enhanced precision, albeit with a concomitant increase in processing time [13]. According to a recent umbrella review, it was concluded that, consequently, AI ought to be perceived at present as an auxiliary instrument that aids, rather than substitutes, clinical judgment. In light of the insights gained from this analysis, AI methodologies are optimally employed as supplementary strategies to augment diagnostic precision, optimize operational processes, and increase the efficacy of dental practice. The use of data augmentation to address class imbalance (successes outnumbering failures) ensures robust training, aligning with best practices in medical imaging AI [14].

This study identified several predictors of single-unit dental implant failure based on preoperative CBCT scans and clinical variables. In the training/validation set, higher preoperative bone density (602.05 ± 148.18 HU in failures vs. 532.05 ± 158.49 HU in successes), wider mesiodistal space at the apex (23.69 ± 5.23 mm vs. 21.16 ± 5.59 mm), shorter implant length (9.81 ± 1.28 mm vs. 10.39 ± 1.42 mm), and higher insertion torque (33.86 ± 2.71 N vs. 32.05 ± 4.48 N) were significantly associated with implant failure. These findings align with prior research indicating that excessive torque and shorter implants (<10 mm) may increase mechanical stress, potentially compromising osseointegration [15,16]. Unlike previous studies that reported smoking as a significant risk factor [17], smoking status in this cohort showed no significant association with failure, possibly due to balanced distribution or sample size limitations. The Mask R-CNN model’s ability to integrate these radiological and clinical features yielded superior predictive performance (accuracy: 0.943, Cohen’s kappa: 0.87), highlighting the advantage of combining imaging-based bone characteristics with clinical parameters for comprehensive risk assessment.

Clinical implications

This AI model could revolutionize preoperative planning by providing reliable risk stratification and enabling tailored surgical protocols, such as avoiding immediate loading in high-risk cases. Reducing the implant failure rate by 3-10% could lower revision costs and improve patient outcomes. Integration into CBCT software can streamline workflows, particularly in resource-limited settings, enhancing access to precision implantology. The model’s visualizations can also support patient communication and training for novice surgeons.

Limitations

The retrospective, single-center design limits the generalizability across diverse populations and implant systems. Another constraint identified was that each dental implant sharing the same foundational system exhibited variations in structure contingent upon its diameter and length. Nonetheless, our dataset does not incorporate these variables. Despite the existence of numerous dental implant systems with diverse designs, only two categories of dental implant systems were represented in the dataset, thus constraining their practical applicability. While de-identified data ensured ethical compliance, prospective multicenter trials are needed to validate the external validity and assess the real-world performance.

## Conclusions

This study demonstrated the efficacy of Mask R-CNN in predicting single-unit dental implant outcomes using preoperative CBCT scans, outperforming both junior and senior implantologists in terms of reliability and discriminative ability. By leveraging automated feature extraction, the model effectively identified critical predictors of implant failure, such as bone density, mesiodistal space, dental implant length, and insertion torque, while mitigating human subjectivity and variability. The model’s integration of radiological and clinical data underscores its potential as a decision support tool, offering a standardized, precise approach to preoperative risk assessment. Future research should focus on prospective, multicenter validation to enhance generalizability and explore applicability in complex surgical scenarios.
